# Carrier Trap Density Reduction at SiO_2_/4H-Silicon Carbide Interface with Annealing Processes in Phosphoryl Chloride and Nitride Oxide Atmospheres

**DOI:** 10.3390/ma16124381

**Published:** 2023-06-14

**Authors:** Ernest Brzozowski, Maciej Kaminski, Andrzej Taube, Oskar Sadowski, Krystian Krol, Marek Guziewicz

**Affiliations:** 1 Łukasiewicz Research Network–Institute of Microelectronics and Photonics, Al. Lotników 32/46, 02-668 Warsaw, Poland; ernest.brzozowski@imif.lukasiewicz.gov.pl (E.B.);; 2 Institute of Microelectronics and Optoelectronics, Warsaw University of Technology, Ul. Koszykowa 75, 00-662 Warsaw, Poland

**Keywords:** SiC, surface states, semiconductor/dielectric interface, NO, POCl_3_

## Abstract

The electrical and physical properties of the SiC/SiO_2_ interfaces are critical for the reliability and performance of SiC-based MOSFETs. Optimizing the oxidation and post-oxidation processes is the most promising method of improving oxide quality, channel mobility, and thus the series resistance of the MOSFET. In this work, we analyze the effects of the POCl_3_ annealing and NO annealing processes on the electrical properties of metal–oxide–semiconductor (MOS) devices formed on 4H-SiC (0001). It is shown that combined annealing processes can result in both low interface trap density (D_it_), which is crucial for oxide application in SiC power electronics, and high dielectric breakdown voltage comparable with those obtained via thermal oxidation in pure O_2_. Comparative results of non-annealed, NO-annealed, and POCl_3_-annealed oxide–semiconductor structures are shown. POCl_3_ annealing reduces the interface state density more effectively than the well-established NO annealing processes. The result of 2 × 10^11^ cm^−2^ for the interface trap density was attained for a sequence of the two-step annealing process in POCl_3_ and next in NO atmospheres. The obtained values D_it_ are comparable to the best results for the SiO_2_/4H-SiC structures recognized in the literature, while the dielectric critical field was measured at a level ≥9 MVcm^−1^ with low leakage currents at high fields. Dielectrics, which were developed in this study, have been used to fabricate the 4H-SiC MOSFET transistors successfully.

## 1. Introduction

An increasing number of mobile and power electronic devices induces demand for modern, fast chargers and power modules. Silicon carbide is a promising material for producing power electronics elements such as Schottky diodes, bipolar transistors, and metal-oxide field-effect transistors (MOSFETs). These elements are applied to converters for various applications [1], ranging from motor drives for different kinds of vehicles [2], high-temperature [3,4,5,6], and high-frequency applications, to renewable energy generation [7,8]. At the present time, SiC power devices offered on the market are junction field-effect transistors (JFETs), metal–oxide–semiconductor field-effect transistors, bipolar junction transistors (BJT), and bipolar transistors with isolated gate (IGBTs). MOSFETs are particularly important due to their application in high-power and high-frequency switching devices. A major bottleneck in developing these transistors is the low level of channel carrier mobility resulting in high series resistance of a channel region. This is the most important problem in power devices, which often require large currents. The increase in channel resistance is mainly caused by electrically active structural imperfections inside the gate dielectric and at the interface between the dielectric and semiconductor, called carrier traps [9,10]. High trap density of the oxide layer is also a serious problem for the application of such dielectrics as a passivation layer in power device structures, for example in junction terminations. All naturally occurring defects, such as interstitial carbon clusters, Si and C vacancies at the SiC surface, and oxygen vacancies in the oxide gate film [9], are responsible for the formation of electrically active traps [10]. The presence of charges ‘trapped’ in such defects in the oxide layer or at the interface strongly influences the transport of the charge carriers in the conduction channel. Previous studies show that effective channel mobility versus gate voltage is influenced by several physical scattering effects [11]. The high density of interface trap states (D_it_) close to the conduction band and charges built in oxide strongly impacts the maximum value of the mobility at low fields. In contrast, the near-interface oxide traps and surface roughness are the main factors affecting the mobility at high fields. The present study focuses on the former problem.

The interface trap states are generated during the formation of the gate oxide film, produced via thermal oxidation of SiC in pure oxygen (dry oxidation) or in oxygen with the addition of water vapor (wet oxidation). Different types of defects are observed in the oxide film depending on temperature and oxidation time, the SiC crystal surface’s orientation, and chemical treatments of the surface before and during the oxidation process [12,13,14,15]. A major source of defects responsible for electrically active trap centers, especially those close to the conduction band edge, are various carbon-related defects such as carbon dimmers and carbon interstitials, as revealed by Density Functional Theory (DFT) simulations [16,17,18]. Some defects can be reduced via nitridation that is expected to lead to the formation of strong Si≡N bonds and passivation of Si dangling bonds at the interface. Moreover, nitridation can help remove C-oxide compounds from the SiO_2_/SiC interface, which is called nitrogen-assisted carbon removal. Such a thermal process in ambient nitrogen is effective only at very high temperatures, as high as 1350 °C or above [19]. Nitridation of SiO_2_/SiC interfaces at lower temperatures was extensively studied in an oxynitride or a nitric oxide environment [20,21,22], but different impacts on the density of interface trap states were noted [23]. The nitridation process not only reduces the D_it_ but can also improve the gate oxide reliability [24]. Experimental works have shown that high-temperature processes at the SiO_2_/SiC interface are complicated, and the results are not easily repeatable on different SiO_2_/SiC structures.

Another way to improve channel charge mobility is with the introduction of phosphorous ions (group V elements) into the SiO_2_/SiC interface via thermal diffusion through the SiO_2_ film in a vapor of the compounds containing the element. It is the thermal P diffusion process with trichlorophosphate (POCl_3_) vapors, known in silicon technology. Some papers confirmed improved channel electron mobility in MOSFET, but the oxide film’s parameters changed after the thermal POCl_3_ process [25,26,27]. Moreover, ions introduced into the oxide film can influence other MOSFET parameters, for example, oxide gate reliability such as dielectric strength, higher leakage at high fields, and thermal instability under high electric fields [28]. Dielectric strength degradation was previously indicated as a weak point of the dielectric layer formation in POCl_3_ processes. Considering that hi-tech equipment available for the high-temperature processes required in SiC technology has some specific physical conditions, every post-oxidation annealing process should be optimized to reach the expected improvement in SiO_2_/SiC interface quality. We chose the post-oxidation processes of thermal annealing in POCl_3_ and nitride oxide atmospheres to verify such treatment of the SiO_2_/SiC-4H interface on the MOS parameters. A few combinations of possible procedures were applied to verify some reported results [29,30,31,32] and recognize the best one. However, new solutions in gate oxide technology try to eliminate SiC oxidation via deposition of SiO_2_ or other dielectric metal oxides [33,34]. Post-deposition thermal annealing processes or plasma treatments are still applied to ensure a perfect dielectric-film/SiC interface [34,35,36]. By lowering the density of interface states, we increase reasonable channel mobility. Consequently, a low ON-resistance of the MOSFET can be attained, which is desirable in power transistors. Both applications require high dielectric strength and low leakage current at high electric fields [37]. The present investigations show that, despite previous concerns about the application of POCl_3_ annealing and oxidation processes, it is possible to realize dielectrics with very low trap state density and high critical fields by using proper technological treatment and a combination of POCl_3_ and NO annealing processes.

## 2. Experimental Data

N-type 4H-SiC (0001) 4° off substrates from Cree Inc. (Durham, NC, USA) (donor concentration *n* = 5 × 10^18^ cm^−3^) with 20 μm thick, n-type-doped epitaxial layer (*n* = 1.1 × 10^15^ cm^−3^) grown by Showa Denko Materials Co., Ltd. (Tokyo, Japan) were used in our experiments. The substrates were cleaned successively in boiling organic solvents of acetone and propanol. Next, they were etched in Piranha solution (H_2_SO_4_:H_2_O_2_:H_2_O, 1:1:1 ratio), rinsed in deionized water, deoxidized in buffered HF solution, and dried in nitrogen blow. Subsequently, for the first series of the samples, high-temperature dry oxidation in an oxygen environment at 1175 °C for 360 or 180 min was conducted. For the second series, wet oxidation in oxygen with water vapors at 1175 °C for 80 min was performed. As a result of these thermal processes, SiO_2_ layers with thicknesses of about 50 or 70 nm were formed on the 4H-SiC samples. Then, specific post-oxidation annealing processes in POCl_3_ and NO environments were performed, followed by annealing in N_2_ for 30 min. POCl_3_ vapor was supplied from a bubbler by using nitrogen flow. The POCl_3_ annealing was conducted at 1000 °C for 10 min. Next, annealing was completed in pure N_2_ at 1000 °C for 30 min or in NO at a temperature of 1000 °C, 1100 °C, or 1175 °C. For chosen samples, annealing in NO at 1000 °C was performed directly after oxidation. Identification symbols of the studied samples are collected in Table 1. Characterization of the Si-oxide film by thickness and refractive coefficient was applied using a spectroscopic ellipsometry technique after every annealing process. The film parameters were calculated based on the fitting of measured ellipsometric parameters using the Sellmeier and Cauchy models of the dielectric film. The next step in MOS fabrication was reactive ion plasma etching using CF_4_/O_2_ plasma (rf power *p* = 100 W, pressure *p* = 50 mT) to remove silicon dioxide from the backside of the samples. Ti/Ni 10/150 nm thick films were deposited via e-beam evaporation technique on the backside for ohmic contact metallization. The ohmic contacts were formed by annealing in the Ar atmosphere at 1050 °C for 3 min. Next, photolithography and a lift-off technique were used to form capacitance structures. A Ti/Al 10/150 nm metallization was fabricated as metallic circles with diameters of 150, 200, 300, and 750 μm. The last annealing of the structures was performed in Ar at 320 °C for 5 min. Electrical methods determining carrier trap density at the oxide/semiconductor interface are based on capacitance versus voltage measurements (C-V) [38,39,40] of the MOS structure. We applied the high–low frequency method (HI-LO), which replaces an ideal characteristic from the high-frequency method (HF) with a low-frequency measured characteristic (LF) or quasi-static characteristic (quasi-static HI-LO method). This further improves the resolution of the method, eliminating a major source of error—a basic HF method uses ideal C-V characteristics for the calculation of (interface trap) MOS capacitance (C_it_), which does not include any fixed or near-interface-trap (NIT) type charges affecting C-V shape during the voltage sweep. The quasi-static HI-LO method depends on applying a frequency with period T higher than for the traps (T > τ_it_) so that every interface trap responds fully to the gate voltage. Then, the interface trap capacitance C_it_ is determined by the formula:

(1) $$C_{it}={\left(\frac{1}{C_{LF}}-\frac{1}{C_{ox}}\right)}^{-1}-{\left(\frac{1}{C_{HF}}-\frac{1}{C_{ox}}\right)}^{-1}$$

and D_it_ can be calculated by:

(2) $$D_{it}=\frac{\Delta C}{q}{\left(1-\frac{C_{HF}+\Delta C}{C_{ox}}\right)}^{-1}{\left(1-\frac{C_{HF}}{C_{ox}}\right)}^{-1}$$

where C_OX_ is oxide capacitance, ΔC = C_LF_ − C_HF,_ C_LF,_ and C_HF_ are normalized capacitances measured at LF or HF, respectively [41].

Our oxidation and annealing procedures were applied to fabricate a gate in a planar 4H-SiC MOSFET device. The base was formed by deep implantation of Al^+^ ions with an energy of 450 keV in the 20 μm thick epitaxial n-SiC layer; source and drain regions were donor-doped to the level of 2–5 × 10^19^ cm^−3^ by narrow implantations of P^+^. After forming a graphite cap layer from a 1 μm thick photoresist film on the implanted samples, thermal activation of the dopants was conducted by annealing in a vacuum at a temperature of 1800 °C for 30 min. The mesa structure was formed with photolithography and ion-reactive ion etching to separate the MOSFETs. Wet oxidation and an optimized sequence of the two-step annealing process in POCl_3_ and next in NO atmospheres were applied for gate SiO_2_ formation. Ohmic contacts were produced from Ti/Ni and Ti/Al/Ti metallization layers to n-type and p-type regions, respectively. In contrast, ohmic contact formation was performed via annealing at 1050 °C for 2 min in Ar. Ti/Al layers were deposited as the gate electrodes. The channel length (L) and width (W) were designed for 5 and 100 μm, respectively. The field-effect mobility (*μ_FE_*) was determined from the MOSFET transconductance measurements. 

## 3. Results and Discussion

**Figure 1:**
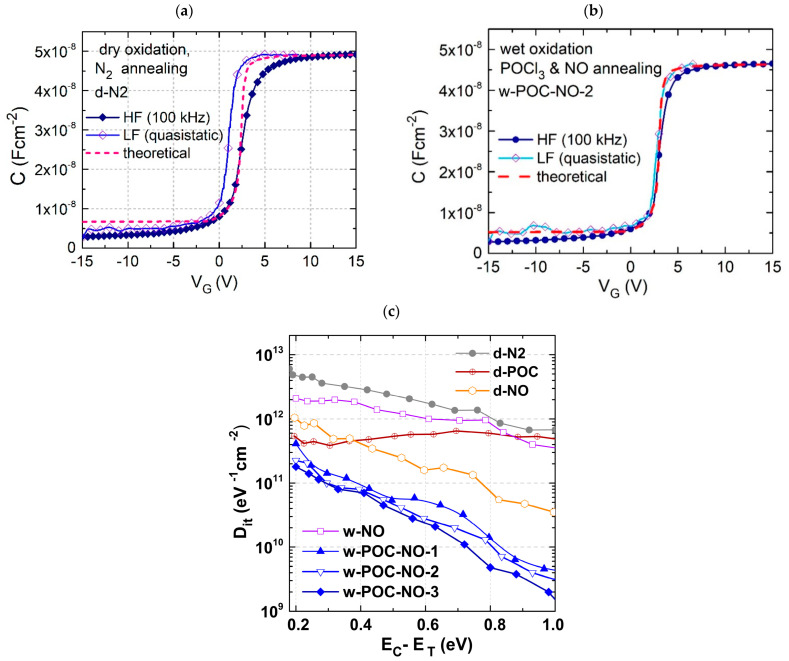
C−V characteristics of MOS SiO_2_/4H-SiC structures formed by using oxide formation processes: dry oxidation and N_2_ annealing—(**a**), successive POCl_3_ and NO annealing—(**b**); interface trap state density D_it_ versus energy distance from conductance band edge calculated for the SiO_2_/4H-SiC structures formed by using dry or wet oxidation, and post-oxidation annealing processes in N_2_, POCl_3_, and/or NO environments—(**c**).

The obtained structures were characterized by current-voltage (I-V), quasi-static, and high-frequency C-V measurements (f = 100 kHz, amplitude of 30 mV), applying polarization from accumulation to depletion and then returning to the accumulation state. Each measurement was preceded by applying a voltage to the sample and forcing accumulation to ensure full trap occupancy at the beginning of the measurement. The length of this initial voltage stress was determined experimentally for each sample by applying stress with increasing duration. The shortest period not resulting in a further C-V characteristic shift was used during measurements. C-V characteristics for dry-oxidized and N_2_-annealed samples are shown in Figure 1a. One can see that quasi-static C-V is shifted to the left with respect to the theoretical curve.

The C-V results for samples after post-oxidation annealing in POCl_3,_ and NO are shown in Figure 1b. The difference between theoretical and measured C-V curves is significantly smaller than for the samples annealed only in N_2_. Such figures correspond with the interfacial trap state density in the samples, especially in the energy range near the Fermi level situated close to the conduction band. The calculated D_it_ vs. energy can be seen in Figure 1c. The D_it_ values obtained for the d-N2 sample are consistent with the previously reported D_it_ values for dry oxidation [36,42,43] and, near the conduction band edge, considerably exceed 1 × 10^12^ eV^−1^cm^−2^. Since the sample was not treated with any processes aimed at reducing trap density, it can be used as a reference sample for comparison with others. 

It is interesting to compare the dry and wet oxidation in terms of trap density. The kinetics of oxidation suggest that wet oxidation is preferable due to the much shorter time required to form gate oxide [44]. Some results suggest that this process can result in lower interface state densities than conventional dry oxidation [45,46]. Recently, it has been shown that, during oxidation, the wet oxidation process generates lower interface stresses [45,47]. Since hydrogen has been shown to passivate dangling bonds at the SiO_2_/SiC interface [48], and due to the amorphous structure of the oxide, those defects appear in a variety of trap energies, mostly located close to the conduction band, so wet oxidation can be used as an efficient and better process for SiO_2_ formation during the oxidation of SiC. Even though our study does not fully confirm this claim, we have been focused on wet oxidation because this process consumes less energy. The wet oxidized sample is characterized by D_it_ (at 0.2 eV below E_C_) with an approximately 30% lower value than that for d-POC.

The interface trap densities D_it_ for the samples after post-oxidation processes are presented in Figure 1c. These are compared for the energy of 0.2 eV below the conduction band and included in Table 2. The trap density D_it_ behavior of the NO-annealed sample is consistent with previous data [20,24,49]. However, the results for the POCl_3_-annealed sample must be discussed. Although it is generally agreed that POCl_3_ post-oxidation annealing improves channel mobility of the MOSFET by reducing density of interface trap states [50], the energy distribution of trap density affected by this process is unclear. Different studies provide dissimilar D_it_ profiles [51,52,53,54,55,56,57] for POCl_3_-treated samples. Pascu et al. showed a little reduction in the trap density near the conduction band edge and almost flat D_it_ profile for deeper levels in the bandgap. However, Okamoto et al. observed that the trap state density near the conduction band edge is reduced by approximately one order of magnitude as compared with a dry-oxidized SiC reference, and it is almost constant for energies deeper in the bandgap. The present study is roughly concurrent with our previously reported findings [27]. This result is also consistent with the theoretical research of Kobayashi et al. [58], which shows via DFT simulations that, at the upper band, close to the conduction band, carbon-related defects play a dominant role in the interface trap density profile [17,59,60,61], and these can be reduced due to the creation of the −O_3_PCO_2_ in the oxide film near the interface during POCl_3_ treatment. A recent study [54], also based on the DFT computation, suggests that phosphorus incorporated near the interface oxide region destabilizes carbon defects, especially carbon dimmers, and some Si defects through structural reconstruction. It was concluded that phosphorus affects mostly traps with energies approximately 0.15–0.4 eV below the conduction band. This is consistent with the curve line shown in Figure 1c for the dry-oxidized sample after the POCl_3_ treatment, designated as d-POC. The D_it_ for the traps with energies below 0.4 eV is reasonably lower than for the reference, and almost unchanged at higher energy.

The best results were obtained by combining the post-oxidation processes in the sequence of POCl_3_ and NO annealing, as seen in Figure 1c. A pronounced slope in the D_it_ profile is visible here for the samples after wet oxidation, POCl_3_ annealing, and successive NO annealing. Moreover, the influence of annealing temperature on the D_it_ profile can be observed, and 1175 °C seems to be the optimal temperature for NO annealing (w-POC-NO-3). The idea behind the sequence was to combine the processes of (1) destabilization of C defects and (2) incorporation of nitrogen into the interface via thermal diffusion. It has been shown [9,16,19] that NO effectively introduces nitrogen into the interface. The mechanism behind improving oxide quality with the annealing process with nitrogen compounds has been theoretically investigated using DFT towards shifting the trap energy associated with a given defect outside the bandgap. In these simulations, carbon-type defects and the dominant defect contributing to the high trap density close to the conduction band edge were considered [62,63,64]. Older as well as newer studies have shown that other possible types of Si-Si bond defects can be removed with nitrogen passivation [65,66,67]. Particularly, NO annealing efficiently removes active states of Si-related defects such as Si-Si bonded atoms, Si dangling bonds, and silicon vacancy [68]. These defects are responsible for introducing trap states nearer the middle of the gap, as was shown by Nakanuma et al. [68]. Numerous Si dangling bonds can be repaired under optimal thermal conditions thanks to the nitrogen reaction with Si bonds at the interface on both sides of the SiC/SiO_2_ interface.

The obtained values of oxide thickness t_ox_, trap surface density D_it_, effective static charge Q_eff_, and flat-band potential V_FB_ parameters of the MOS structures are collected in Table 2. Different film thicknesses are the results of different thermal and gas conditions. Ellipsometry measurements of the wet-oxidized and POCl_3_-annealed films are characterized by a thickness in the range of 73.9–86 nm and refractive index *n* = 1.489, which is increased in relation to the n-index of the model of thermal SiO_2_ layer on Si. An increase in thickness of approximately 20 nm or a bit above was registered for the dry or wet oxide films after the POCl_3_ annealing process. Such a rapid oxidation process may be partially responsible for the effective removal of carbon defects from the interface, as previously reported in [27,31,50]. This can be explained by the transformation of the oxide layer into phosphosilicate glass (PSG) during annealing. In our experiment, the process temperature was kept at a relatively low level (1000 °C), so the transition rate to PSG was slower than reported in other works. The oxygen–SiC reaction at this temperature is too slow for efficient SiO_2_ formation in the timescale used in the experiment. However, it is worth mentioning that the SiC oxidation reaction in the POCl_3_ ambient has been reported to have lower activation energy than in the dry oxygen [27,69].

**Figure 2:**
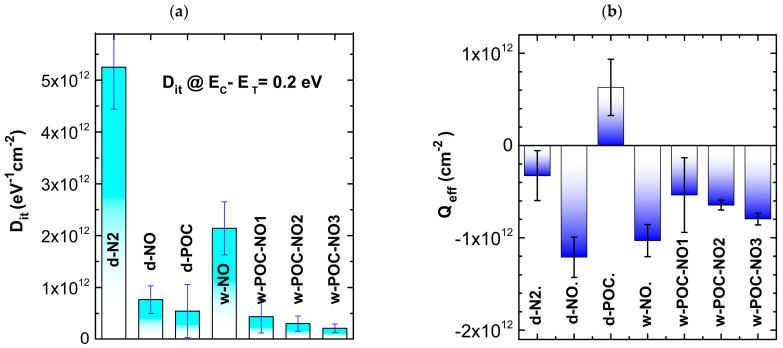
Interface trap state density Dit at E_C_ − E_T_ = 0.2 eV obtained for gate oxide thermally formed on 4H-SiC(0001) at different atmospheres and post-oxidation annealing processes—(**a**), surface charge density Q_eff_ measured on the MOS structures—(**b**).

The interface trap density D_it_ at E_C_ − E_T_ = 0.2 eV obtained for the SiO_2_/4H-SiC samples annealed at different atmospheres and temperatures is illustrated in Figure 2a, while an effective surface charge density (Q_eff_) is shown in Figure 2b. The most important observation that can be drawn from these results is a change in the value and sign of the effective charge value and in the sign for the sample annealed only in POCl_3_ (d-POC). These can be attributed to the formation of phosphosilicate glass (PSG) in the volume of the oxide layer, where P-oxide is built into the layer, and such formation has been described earlier [53]. The increased positive effective surface charge suggests a high phosphorus concentration near the interface region, most likely achieved due to the annealing process at a temperature similar to those in experiments described by others [50,51,52,53]. The chemical reaction of POCl_3_ with SiC at 1000 °C is too slow for quick oxide formation in the timescale used in our experiment. Therefore, a higher phosphorus concentration can be achieved through the diffusion process [57]. The POCl_3_ interaction with SiC at the interface can be the cause of a significant reduction in the interface trap state density close to the conduction band. Moreover, the samples after POCl_3_ treatment and subsequently annealed in NO at a higher temperature are characterized by the effective surface charge density (Figure 2b) close to that observed for the reference sample annealed only in N_2_.

On the samples annealed in POCl_3_, it was also observed that after annealing in the NO atmosphere, the thickness of the oxide layers slightly decreased. The effect was measured with ellipsometry on additional wet-oxidized SiC samples. Results are presented in Table 3. The difference in thickness (Δt_ox_) increases with NO annealing temperature, while the n-index is still high. The higher temperature causes an amplified effect on the process. Such decrease in thickness may be caused by the evaporation of some P-oxide compounds from the dielectric layer at the applied temperature. 

Based on the C-V measurements, calculation of the interface trap density, and oxide thickness measurements, we suggest that the subsequent NO annealing process performed after the POCl_3_ process can reverse some of the changes in the oxide structure initially created by phosphorus compounds. It leads to a decrease in P concentration in the layer and, consequently, to a decrease in the positive charge accumulated in this layer during POCl_3_ annealing. Formation of the PSG-like layer during POCl_3_ annealing can be the most critical step in the application of such a layer to a gate dielectric in MOSFETs, which has been studied in the last decade [25,26,27,28]. Such considerations are mainly due to two reasons: (1) it was shown that introducing phosphorus can decrease the energy barrier for the Fowler–Nordheim current that dominates the conduction mechanism at the high electric field leading to the premature dielectric breakdown [56], and (2) a high concentration of phosphorus ions in the oxide film can lead to thermal instability of the device, as some of the ions in the oxide volume can be mobile under the strong electric fields present in the MOS device during its operation, modulating the device’s threshold voltage.

**Figure 3:**
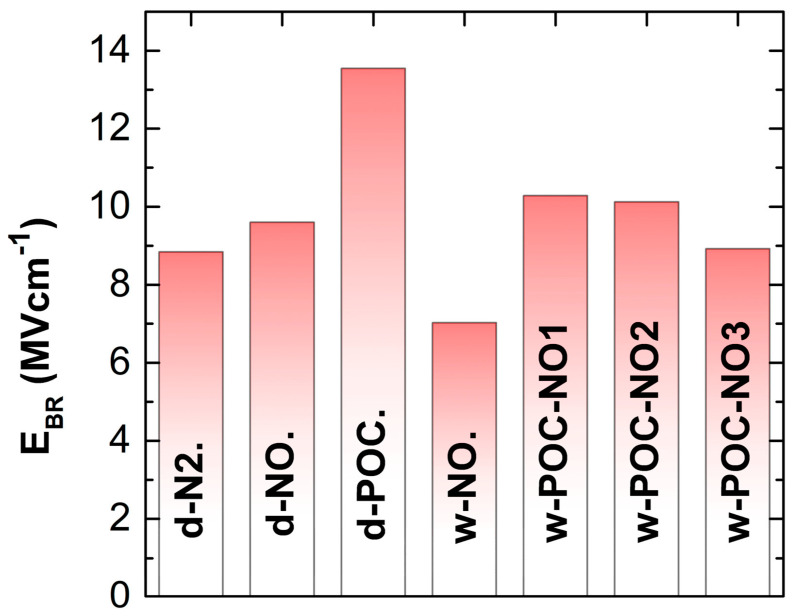
Breakdown electric field measured on gate oxide thermally formed on 4H-SiC(0001) at different atmospheres and post-oxidation annealing processes.

The low-temperature NO annealing step developed here can reduce both problems by maintaining the desired low interfacial trap density. To support this claim, we have measured the breakdown electric field for our samples, and the results are shown in Figure 3. The critical field for the dry oxides annealed successively in N_2_ or NO was near 10 MVcm^−1^. A 30% lower critical field characterized the wet-oxidized structures. This drift has been previously observed [19]. On the other hand, the critical field for oxides annealed in the phosphoryl chloride atmosphere was above 10 MVcm^−1^. This result is better than reported for high-temperature annealing [56], and it is concurrent with the results reported by Pascu et al. [70], taken on SiO_2_/SiC samples after the POCl_3_ annealing process at a similar temperature. The processes applied for reducing the density of the trap states do not significantly affect the breakdown voltage resistance of the oxide films. Annealing processes in the phosphoryl chloride atmosphere followed by a nitric oxide atmosphere at 1175 °C allow obtaining the critical field of ~9 MVcm^−1^. Moreover, the MOS structures with this two-step annealed dielectric are characterized by a very low leakage current density, <3 × 10^−7^ Acm^−2^, at an applied stress voltage of −100 V.

The successful application of the dielectric layer annealed in accordance with the developed procedure was confirmed by electrical measurements of the manufactured planar MOSFET transistors with a gate width of 100 µm, which showed the field effect mobility *μ_FE_* of the channel electrons at the level of 30 cm^2^V^−1^s^−1^. Such a high mobility is consistent with the results published in the review work for MOSFETs with the dielectric gate passivated via NO annealing at high temperature. This confirms the usefulness of the proposed procedures for the thermal formation of SiO_2_ dielectrics in SiC MOSFETs. Some papers present μ_FE_ mobility with a peak value close to 100 cm^2^V^−1^s^−1^ [25,26,29], suggesting the possibility of further improvement of the channel mobility with additional optimization of technological processes during fabrication of our MOSFET structure, for example by better recovering the mono-crystalline structure of the implanted p-well region. The obtained results are consistent with other studies in this matter, which have been successively published over the last 20 years [18], until now [68,71].

## 4. Conclusions

MOS SiO_2_/4H-SiC structures were characterized using C-V measurements and ellipsometry techniques. It was pointed out that two-stage annealing of SiO_2_/4H-SiC structures in POCl_3_ atmosphere at 1000 °C for 10 min and subsequently in NO atmosphere at 1100 °C or 1175 °C reduced the interface trap state density D_it_ down to the level of 2 × 10^11^ cm^−2^ in the vicinity of the conduction band edge. The reduction in the trap state density is related to the specific phosphorous impact on carbon-associated trap states at the SiO_2_/SiC interface and to nitrogen attaining and passivating the interface traps. The nitridation process of NO annealing at a temperature of 1100 °C or higher is more effective in combination with phosphorous inclusions in the oxide film. The results show that, by performing two-step annealing at a temperature lower than the threshold value for efficient SiC oxidation in POCl_3_ and subsequently in NO, one can obtain the dielectric layer that has both low interface state density and high critical electric field, such as the one obtained via simple dry oxidation (the critical field of ~9 MVcm^−1^). Additionally, it was observed that those layers have very low leakage current (<3 × 10^−7^ Acm^−2^ at an applied stress voltage of −100 V), making them suitable for application in SiC device technology. Such layers can be used in high field conditions, where high breaking voltage and small leakage currents are required, for example, as surface passivation for junction termination extension structures, in passivation of SiC IGBT structures, or possibly as gate oxides for MOSFET and IGBT devices.

## Figures and Tables

**Table 1:** Parameters of the thermal process used for thermal formation of SiO_2_/4H-SiC structures (oxidation temperature of 1175 °C) and the identification symbols (ID) of the studied samples. Post-oxidation annealing processes in POCl_3_, NO, and N_2_ environments were performed at temperature T for time intervals t of 10 min, 30 min, and 30 min, respectively.

Process Type, Parameter	Values						
Oxidation, Time *t* (min)	Dry	Dry	Dry	Wet	Wet	Wet	Wet
	360	180	360	80	80	80	80
POCl_3_ T (°C)	-	-	1000	-	1000	1000	1000
N_2_ T (°C)	1000	-	1000	-	1000	1000	1000
NO T (°C)	-	1000	-	1000	1000	1100	1175
sample identification, ID	d-N2	d-NO	d-POC	w-NO	w-POC-NO-1	w-POC-NO-2	w-POC-NO-3

**Table 2:** Parameters of the MOS structures obtained from C−V measurements after thermal processes used to develop SiO_2_/4H-SiC interface: oxide film thickness t_ox_, trap surface density D_it_ calculated for energy near the conduction band level E_C_ − E_T_ = 0.2 eV, and flat band potential V_FB_.

Parameter	Sample Identification, ID						
	d-N2	d-NO	d-POC	w-NO	w-POC-NO-1	w-POC-NO-2	w-POC-NO-3
t_ox_ (nm)	80	50	78	53	58	73	73
D_it_ × 10^11^ (eV^−1^cm^−2^) (E_C_ − E_T_ = 0.2 eV)	48	7.6	5.4	22	4.5	3.1	2.0
V_FB_ (V)	2.37	3.97	−1.15	3.52	2.47	3.32	3.82
Q_eff_ × 10^11^ (cm^−2^)	−3.2	−12	6.3	−10	−5.4	−6.5	−7.9

**Table 3:** The thickness t_ox_ of oxide films formed by wet oxidation and POCl_3_ annealing, just before and after NO annealing at different temperatures T_NO_; refractive index η (at λ = 650 nm) and thickness difference Δt_ox_. The t_ox_ and η-index calculated based on ellipsometry measurements.

Before NO	Temperature	After NO		
t_ox_ (nm)	T_NO_ (°C)	t_ox_ (nm)	η (λ = 650 nm)	Δt_ox_ (nm)
75	1000	66.4	1.472	−8.6
75	1100	62.1	1.487	−12.7
86	1175	72.6	1.488	−13.4

## Data Availability

The data presented in this study are available on request from the corresponding author. The data is not publicly available due to internal regulations.
